# Stress and Maladaptive Coping of Italians Health Care Professionals during the First Wave of the Pandemic

**DOI:** 10.3390/brainsci11121586

**Published:** 2021-11-30

**Authors:** Paolo Grandinetti, Martina Gooney, Florian Scheibein, Roberta Testa, Gaetano Ruggieri, Paolo Tondo, Anastasia Corona, Graziella Boi, Luca Floris, Valerio F. Profeta, John S. G. Wells, Domenico De Berardis

**Affiliations:** 1 Department of Territorial Assistance, ASL Teramo, 64100 Teramo, Italy; grandinetti.paolo@gmail.com (P.G.); gaetano.ruggieri@aslteramo.it (G.R.); paolo.tondo@aslteramo.it (P.T.); 2 School of Health Sciences, Waterford Institute of Technology, X91 K0EK Waterford, Ireland; mgooney@wit.ie (M.G.); florian.scheibein@wit.ie (F.S.); jswells@wit.ie (J.S.G.W.); 3 Department of Mental Health, ASL Teramo, 64100 Teramo, Italy; robytesta@gmail.com (R.T.); valerio.profeta@aslteramo.it (V.F.P.); 4 Department of Mental Health and Addiction, ATTS Sardegna-Zona Sud, 09100 Cagliari, Italy; anastasia.corona@atssardegna.it (A.C.); graziella.boi@atssardegna.it (G.B.); luc.floris@atssardegna.it (L.F.)

**Keywords:** on-line behaviours, coping behaviours, maladaptive coping, stress, anxiety, healthcare management, pandemic, stress and COVID-19

## Abstract

Stress during the pandemic has had an impact on the mental health of healthcare professionals (HCPs). However, little is known about coping and “maladaptive” coping behaviours of this population. This study investigates “maladaptive” coping behaviours and their correlation with stress, anxiety and insomnia of Italian HCPs during the pandemic. It reports on a cross-sectional, descriptive and correlational study based on a survey of 1955 Italian HCPs. Overall participants reported increases in cigarette smoking, time spent online and video playing. Overall reported alcohol consumption decreased but increased in those reporting drinking more than once a week. Those reporting starting smoking during the pandemic were found to have higher SAS and PSS scores. Those reporting being online for 3 or more hours were found to have higher ISS scores. Doctors who reported playing video games were found to have higher PSS, ISS and SAS scores whilst nurses who reported playing video games were found to have higher ISS scores. Doctors who reported playing for longer than one hour had higher PSS scores. Online behaviours may be a coping behaviour of HCPs affected by the pandemic. However, this is an underexplored area for the wellbeing of HCPs. These deficits need to be addressed going forward.

## 1. Introduction

Since the identification of the SARS-CoV-2 as a significant public health issue in January 2020 several studies have reported the mental health impacts of the COVID-19 pandemic on the general population and health care professionals (HCPs) focusing largely on depression, anxiety and post-traumatic stress symptoms [1,2,3,4,5,6,7,8].

As outlined by Holmes and colleagues, in stressful circumstances, such as the pandemic, anxiety, depression and harmful behaviours can increase with changes in sleep and lifestyle affecting mental health and stress responses. Thus, social and personal resources can be important factors related to resilience in mitigating stress [9]. Moreover, a good stress response and positive appraisal, specifically of the consequences of the Corona crisis, may be protective against pandemic related stress [10].

Collaboration in health care has been shown to improve patient outcomes and lead to better care for COVID-19 patients and to provide benefits to health care professionals, including reducing extra work and increasing job satisfaction [11,12]. It is also known that some behaviours can be important indicators of stress and psychological health particularly in relation to maladaptation [13].

Several studies have explored the coping strategies of Italian HCPs during the pandemic, showing that Italian emergency healthcare professionals maintained a task-oriented pattern of response following the first wave of COVID-19, with less represented emotional coping strategies [14] and a positive attitude towards the stressful situation being found to be a strong protective factor, while female gender, seeking social support, avoidance strategies and working with COVID-19 patients were found to be considerable risk factors [15]. Vagni and colleagues showed that health care workers were exposed to a large degree of stress and could experience secondary trauma, greater levels of emergency stress and arousal and were more willing to use problem-focused coping. Moreover, individual efficacy in stopping negative emotions and thoughts could have been a protective strategy against stress and secondary trauma [16].

It is known that pandemic-related stress can lead to maladaptive coping behaviours in healthcare professionals [17,18]. However, there appear to be few studies that have explored maladaptive coping strategies used by HCPs whilst caring for patients in the first wave of the pandemic with a particular lack of studies on online dependence and behaviours.

Italy was the first country in Europe to be significantly affected by the first wave of COVID-19, which, at the time, was described as the most serious crisis to affect the country since 1945 [19].

This paper reports on a survey of Italian HCPs’ alcohol consumption, smoking, gambling, hours spent online and videogaming behaviour conducted during the first wave of the pandemic between the 25 April and 3 June 2020.

### 1.1. First Wave of COVID-19 in Italy

The first confirmed Italian case of COVID-19 infection was on 31 January 2020 with the first attributed death occurring on 21 February 2020. The north of the country, Lombardy and Veneto, stood at the epicentre of the Italian COVID-19 crisis. The rest of the country, particularly the South, was less impacted by the pandemic than the North. On 12 March 2020, the Italian Government declared a national lockdown, which ended in June 2020 (Italian Government 2020). Hospitalisations and deaths rapidly increased, overwhelming hospital services, with the peak of the first wave of the pandemic occurring in March and April 2020. In order to deal with the rapid rise in hospitalisations, Italian clinicians were reassigned duties and others came out of retirement to bolster the number of available health care personnel [19]. There was a gradual decrease in hospital admissions and excess deaths from May onwards [20].

From early March 2020, Italian health care personnel reported through social media how they felt overwhelmed and physically exhausted in dealing with the influx of patients and rising COVID-related deaths [21]. Levels of stress, particularly among nurses, were reported as very high along with several suicides being reported [22].

### 1.2. Background to This Study

Dealing with epidemics has been positively correlated with high rates of alcohol and other substance use among HCPs [17]. A study of the impact of the pandemic on the well-being of 695 Italian medical doctors found that 14.2% of the participants reported an increase in alcohol consumption and 43.6% of doctors reported an increase in the number of cigarettes smoked during the day [16]. Notably, three comparative domains of behaviour that have not been studied specifically in healthcare workers during the pandemic are gambling, videogaming and time spent online, especially if associated to maladaptive coping (for example, smoking and alcohol consumption). It is established within the literature that these types of behaviours can become highly addictive and disrupt normal activities of living [17]. There are many cognitive neuroscience studies to support this hypothesis [23,24,25]. Garofalo and colleagues suggest that individual differences in learning style and cognitive abilities (i.e., working memory capacity) play a role in the predisposition to such maladaptive implications [26]. Thus, inappropriate and maladaptive behaviours might potentially show the characteristics of addiction.

This study, therefore, reports on five domains of behaviour that can be addictive in relation to maladaptive coping: gambling, videogaming, time spent online, smoking and alcohol consumption as a consequence of insomnia, stress and anxiety among Italian health workers during the first wave of the pandemic.

## 2. Materials and Methods

### 2.1. Study Design

This paper reports a cross-sectional, descriptive and correlational study based on a survey conducted from 25 April to 3 June 2020. This research as part of epidemiological research conducted by the Italian health authority ASL Teramo.

### 2.2. Participants

This survey of HCPs was a subset of a larger survey of the general population during the COVID 19 crisis in Italy promoted through social media, e-mail, newspapers and ASL Teramo and ATTS Sardegna-zona sud official website. All frontline health care workers were eligible to participate in this study. Although the survey was open to all Italian healthcare professionals, the fact that the survey promoted mainly by ASL Teramo and ASL Sardegna-zona sud probably led to a higher proportion of responses from those territories. Out of a total of 1955 health care professionals from across Italy who completed the survey, 1709 (87.4%) were from the South (including islands). As workers were being surveyed in relation to their maladaptive behaviours, it was decided not to gather data on which unit they worked, to increase privacy and confidentiality and protect the anonymity of survey participants.

### 2.3. Measurements and Outcomes

Sociodemographic data included: age, gender and number of people living in the same household. HCPs’ anxiety, perceived stress and insomnia, at the time of the survey (during the lockdown), were assessed using the Italian versions of the following rating scales:

(1) The 20-item Zung’s Self-Rating Anxiety Scale (SAS) [27] to examine emotional and physical symptoms of anxiety. The total scores ranged from 25 to 100, with ≥45 indicating minimal to most extreme anxiety (Cronbach’s alpha value = 0.82 in this study).
(2) The 10-item Perceived Stress Scale (PSS) [28] to measure the perception of stress. The total scores ranged from 0 to 40, with ≥14 indicating moderate to high stress. (Cronbach’s alpha value = 0.85 in this study).
(3) The 7-item Insomnia Severity Index (ISI) [29] to assess the severity of both night-time and daytime components of insomnia. The total scores ranged from 0 to 28, with ≥8 indicating subthreshold to severe insomnia. (Cronbach’s alpha value = 0.89 in this study).

Participants were also asked to report on a range of variables, including alcohol consumption, smoking cigarettes, time spent playing games requiring the use of money, amount of time spent online and playing video games before and during the lockdown.

### 2.4. Data Analysis

Data were analysed using IBM SPSS statistical software version 25.0. Descriptive statistics were used to summarise sociodemographic, behavioural and mental health outcomes (SAS, PSS and ISI). The χ^2^ test was used for differences between sociodemographic and behavioural variables. The scores of the 3 measurement tools were not normally distributed despite the large sample size and therefore non-parametric tests were used to analyse the data which are presented as medians with interquartile ranges (IQRs). The exact McNemar’s test was used to compare differences in reported behaviours before and after the 11 March. The Mann–Whitney U test was used to examine the difference in mental health outcomes between sociodemographic and other behavioural variables in each of the 3 HCPs. Spearman rank order correlation was used to examine the relationship between anxiety, perceived stress and insomnia. and all other continuous variables. *p* values of less than 0.05 were considered statistically significant.

## 3. Results

### 3.1. Sociodemographic, Anxiety, Stress and Insomnia Profile

Sociodemographic data, behaviours and mental health data are reported in Table 1, Table 2, Table 3 and Table 4. Anxiety was reported by 173 (21.9%) doctors, 185 (33.9%) nurses and 128 (23.8%) other HCPs; 423 (52.3%) doctors, 282 (50.6%) nurses and 252 (46.1%) other HCPs reported stress; 317 (38.3%) doctors, 250 (44.2%) nurses and 203 (36.2%) other HCPs reported insomnia. The majority of respondents were female across all groups: 64.7% of doctors, 79% of nurses and 72.1% of other HCPs. Nurses presented the highest median SAS score of 40 (IQR = 11) followed by other HCPs with a score of 39 (IQR = 9) and doctors with a score of 38 (IQR = 10). Both doctors and nurses reported a median PSS total score of 14 (IQR = 10 and IQR = 9, respectively) whilst other HCPs reported a lower median total score of 13 (IQR = 9). Nurses also presented with the highest median ISI score of 6 (IQR = 9) whilst both doctors and other HCPs reported a median total ISI score of 5 (IQR = 8) (Table 1). A statistically significant difference in all three scales (SAS, ISS and PSS) was found between males and females (*p* = 0.000). There was a negative correlation between age and the SAS (*p* = 0.001), PSS (*p* = 0.000)) and ISI (*p* = 0.006), with low levels on each scale associated with an increase in age (Table 4).

### 3.2. Alcohol

Alcohol consumption was reported to decrease significantly post lockdown in each of the three groups (*p* = 0.000). In all categories alcohol was reported to decrease except for the category “more than once a week”—for medical doctors and nurses. However, the total number of affected individuals was small. One hundred and seventy-seven (21.4%) doctors reported drinking alcohol more than once a week before the lockdown and this increased to 188 (22.7%) during the lockdown. Sixty-two (11%) nurses reported drinking alcohol more than once a week before the lockdown and this increased to 67 (11.9%) during the lockdown. In contrast, for other HCPs, this category decreased from 107 (19.1%) before to 105 (18.8%) during the lockdown.

### 3.3. Smoking

There was no change in the number of people reporting smoking before and during the lockdown. However, a statistically significant increase in the number of cigarettes smoked from before to during the lockdown (*p* = 0.000) was reported in all three professions. Doctors and nurses reporting smoking during the lockdown had significantly higher median PSS scores (*p* = 0.046; *p* = 0.019 respectively) with doctors also reporting significantly higher median anxiety scores than those that did not (*p* = 0.007). In relation to number of people in the household, nurses were the only profession that showed significant differences in terms of smoking. Nurses who reported smoking reported a significantly lower number per household (1.69 ± 1.19) than those that did not smoke (2.05 ± 1.25; *p* = 0.002).

### 3.4. Hours Spent Online

A greater proportion of all three groups reported spending 3 h or more online during the lockdown (*p* = 0.000). Doctors that reported spending more than 3 h online had a significantly higher median ISS score (*p* = 0.000). This was also found in relation to nurses (*p* = 0.006) and other HCPs (*p* = 0.023). Doctors who spent up to 2 h online had lower median SAS scores (*p* = 0.000). An independent *t*-test also revealed that those that spent 3 h or more online were significantly younger in all three professions (*p* = 0.000). Nurses with lower number per household were found to be more likely to report spending 3 h or more online (*p* = 0.032).

### 3.5. Online Gaming

The percentage of each profession playing video games did increase; however, there was no statistically significant difference in the proportion playing video games before and during the lockdown. Those reporting playing video games for 1 h or more increased significantly among other HCPs (*p* = 0.000), nurses (*p* = 0.001) and doctors (*p* = 0.011). In all three groups, those of younger age were more likely to report playing video games. Doctors that reported playing video games had significantly higher PSS (*p* = 0.001), ISS (*p* = 0.002) and SAS (*p* = 0.022) scores and those who reported playing for longer than one hour were also more likely to have higher ISS scores (*p* = 0.024). Nurses who reported playing video games also had higher median PSS scores (*p* = 0.025). In all three groups, the proportion playing paid games decreased significantly (*p* = 0.000). Nurses reporting playing paid games reported higher PSS scores (*p* = 0.009) and SAS scores (*p* < 0.008). The difference was also significant for other HCPs (*p* = 0.04) for SAS scores.

## 4. Discussion

Our levels for anxiety, insomnia and stress are similar to the pooled prevalence of 23.2% for anxiety and 34.3% for insomnia reported in a systematic review by [30] and the prevalence of stress reported by [31] in China (55.1%). Similar to other studies, we found differences in age, gender and occupation for these variables [30,31,32]. However, our results indicate lower levels of severe forms of anxiety and stress. This may reflect the fact that the majority of our sample was drawn from South Italy, which at the time of data collection was less impacted by the pandemic than the North Italy. Therefore, our results for stress, anxiety and insomnia can be seen as a normal response to what was being observed through the media and experienced at the bedside; this could suggest expanding on studies relating to the cognitive regulation of emotional processes [33,34,35].

Limitations of our study include that the majority of the population studied, were based in Southern Italy and the islands and that we did not differentiate between frontline and non-frontline workers. The North of the country was disproportionately impacted by COVID-19 during the first wave of the pandemic and other authors have identified the negative impacts of working directly with COVID-19 patients on HCPs mental health [36]. However, we observed a range of maladaptive behaviours amongst the HCPs studied indicating a range of stresses and impacts as a result of the pandemic.

Future studies of pandemic-related stress and coping in HCPs should take in account both the differences between frontline and non-frontline workers as well epidemiological indicators of exposure to the pandemics. Although the number of respondents reported that their alcohol consumption had decreased, the number of doctors and nurses who reported drinking more than once a week marginally increased. However, there was a statistically significant increase in the reported number of cigarettes smoked in all three groups. Doctors and nurses who reported smoking had higher PSS scores. Nurses reporting a lower number of people per household and medical doctors with higher anxiety were also found to be more likely to smoke. Higher reported anxiety among doctors maybe reflective of a sense of responsibility in relation to accountability for the medical management of cases in a context of a growing medical challenge in which clarity of effective treatment in the first wave was limited [37,38].

In relation to online behaviour, unsurprisingly, younger professionals reported spending more time online than their older participants. We also found a link between those reporting spending more time online (>3 h) and ISS, SAS and PSS scores. Doctors that reported spending more than 3 h online had a significantly higher median ISS score, SAS score and PSS score. Nurses and other HCPs also had significantly higher PSS scores. Our results would suggest that certainly in relation to younger healthcare personnel greater attention needs to be paid towards the occupational health implications of their online habits when dealing with ongoing emergencies and their stressors, both physical and mental.

Younger age was associated with video game playing in all three groups along with being a male doctor. Those who reported playing video games had a higher stress score than those that did not—a relationship which was particularly significant for doctors and nurses. Insomnia was also worse for doctors that reported playing more than one hour online. Reports of playing paid games decreased. HCPs that reported playing paid games had higher anxiety, but this was particularly true for nurses.

Online behaviour particularly in relation to video gaming and paid games appears to be linked to higher stress, anxiety and insomnia levels. These behaviours are under explored with reference to all HCPs in terms of their clinical performance. In the context of the stressors within the pandemic [30], time spent online appears to reflect higher levels of anxiety, stress and or insomnia. Whether or not time spent online gaming contributes to these would need to be further explored.

## 5. Conclusions

Sudden outbreaks of the pandemic disease such as COVID-19, are events that health services will faced for the medium- and long-term future [39]. The challenge for health professionals will be to manage the extensive impacts such outbreaks will pose for them in both their professional and personal lives. Our study indicates that one behavioural expression of stress, and possibly a contributor to that stress, is their engagement with online activity, particularly amongst a generation that has grown up in an online environment. Yet this is a relatively under-explored area for the well-being of health care professionals and not addressed during their undergraduate training as this relates to their well-being. Our results suggest that these deficits need to be addressed going forward into the future.

## Figures and Tables

**Table 1:** Sociodemographic and mental health outcomes during the first wave (post 11 March).

Sociodemographic Variables	Doctor (n = 829)		Nurse (n = 566)		Other HCP (n = 560)	
Sociodemographic Variables	Median (IQR)	n (%)	Median (IQR)	n (%)	Median (IQR)	n (%)
Age	53 (20)		50 (15)		50 (9)	
Female		536 (64.7%)		447(79%)		404 (72.1%)
Male		293 (35.3%)		119 (21%)		156 (27.9%)
Number in household	2 (2)		2 (2)		2 (2)	
**Anxiety (SAS)** ^a^	38 (10)		40 (11)		39 (9)	
Normal		619 (78.1%)		361 (66.1%)		410 (76.2%)
Minimal to severe		173 (21.9%)		185 (33.9%)		128 (23.8%)
**Stress(PSS)** ^b^	14 (10)		14 (9)		13 (9)	
Low		386 (47.7%)		275 (49.4%)		295 (53.9%)
Moderate to high		423 (52.3%)		282 (50.6%)		252 (46.1%)
**Insomnia(ISI)** ^c^	5 (8)		6 (9)		5 (8)	
Normal		512 (61.7%)		315 (55.8%)		357 (63.7%)
Subthreshold to severe		317 (38.3%)		250 (44.2%)		203 (36.2%)

Some percentages calculated using denominator less than stated total due to missing data. ^a^ SAS, Zung’s Self-Rating Anxiety Scale; ^b^ PSS, Perceived Stress Scale; ^c^ ISI, Insomnia Severity Index.

**Table 2:** Sociodemographic and behavioural variables during the first wave (post 11 March).

Sociodemographic Variables	Alcohol Consumption		Smoking		Gambling		Video Games	
	n (%) Mean (SD)	*p* Value	n (%) Mean (SD)	*p* Value	n (%) Mean (SD)	*p* Value	n (%) Mean (SD)	*p* Value
**Medical Doctors**								
Gender †								
Male	234 (80)	0.000	47 (16)	1.000	18 (6)	0.012	74 (25)	0.019
Female	351 (66)		85 (16)		13 (2)		97 (18)	
Age (years) ‡								
Yes	49.34 (11.83)	0.002	48.00 (11.72)	0.023	54.81 (10.76)	0.019	46.41(11.20)	0.000
No	52.00 (11.15)		50.53 (11.65)		49.94 (11.70)		51.09 (11.62)	
**Nurses**								
Gender †								
Male	96 (81)	0.000	33 (28)	0.717	14 (12)	0.017	38 (32)	0.063
Female	230 (52)		114 (26)		23 (5)		103 (23)	
Age (years) ‡								
Yes	48.06 (9.77)	0.308	46.89 (9.97)	0.255	45.00 (10.30)	0.088	45.16 (10.60)	0.001
No	47.20 (10.20)		47.98 (9.95)		47.88 (9.91)		48.54 (9.59)	
**Other HCPs**								
Gender †								
Male	130 (83)	0.000	34 (22)	0.357	9 (6)	0.667	36 (23)	1.000
Female	245 (61)		105 (26)		18 (5)		95 (24)	
Age (years) ‡								
Yes	47.66 (11.41)	0.109	46.45 (11.361)	0.035	49.59 (10.31)	0.496	45.54 (11.52)	0.003
No	49.21 (10.39)		48.74 (10.97)		48.10 (11.16)		48.97 (10.86)	

Some percentages calculated using denominator less than stated total due to missing data. † χ^2^ test; ‡ Independent two-sample *t*-test.

**Table 3:** Behaviours before and during the first wave (post 11 March).

	Before the 11 March n (%)	After the 11 March n (%)	*p* Value (McNemar Test)
Alcohol consumption			
Medical Doctors			
Yes	632 (76%)	585 (71%)	0.000
No	197 (24%)	243 (29%)	
**Nurses**			
Yes	370 (65%)	326 (58%)	0.000
No	196 (35%)	240 (42%)	
**Other HCPs**			
Yes	416 (74%)	375 (67%)	0.000
No	144 (26%)	185 (33%)	
**Smoking**			
**Medical Doctors**			
Yes	133 (16%)	132 (16%)	1.000
No	696 (84%)	697 (84%)	
**Nurses**			
Yes	142 (25%)	147 (26%)	0.267
No	424 (75%)	419 (74%)	
**Other HCPs**			
Yes	141 (25.2%)	139 (24.8%)	0.754
No	419 (74.8%)	421 (75.2%)	
**Gambling**			
**Medical Doctors**			
Yes	80 (10%)	31 (4%)	0.000
No	749 (90%)	798 (96%)	
**Nurse**			
Yes	67 (12%)	37 (7%)	0.000
No	499 (88%)	529 (93%)	
**Other HCP**			
Yes	70 (13%)	27 (5%)	0.000
No	490 (87%)	533 (95%)	
**Hours Spent Online**			
**Medical Doctors**			
Up to 2 h	634 (77%)	454 (55%)	0.000
3 h or more	194 (23%)	374 (45%)	
**Nurse**			
Up to 2 h	424 (75%)	325 (57%)	0.000
3 h or more	142 (25%)	241 (43%)	
**Other HCP**			
Up to 2 h	419 (75%)	301 (54%)	0.000
3 h or more	141 (25%)	259 (46%)	
**Video Games**			
**Medical Doctors**			
Yes	158 (19%)	171 (21%)	0.099
No	671 (81%)	658 (79%)	
**Nurse**			
Yes	132 (23%)	141 (25%)	0.176
No	434 (77%)	425 (75%)	
**Other HCP**			
Yes	121 (22%)	131 (23%)	0.134
No	439 (78%)	429 (77%)	
**Hours Spent on Video Games**			
**Medical Doctors**			
<1 h	150 (77%)	125 (65%)	0.011
≥1 h	46 (23%)	68 (35%)	
**Nurses**			
<1 h	116 (69%)	94 (56%)	0.001
≥1 h	52 (31%)	73 (44%)	
**Other HCPs**			
<1 h	95 (67%)	65 (46%)	0.000
≥1 h	47 (33%)	76 (54%)	

Some percentages calculated using denominator less than stated total due to missing data.

**Table 4:** Difference in mental health scales between various sociodemographic and addiction related variables.

Sociodemographic Variables	Anxiety (SAS) ^a^		Perceived Stress (PSS) ^b^		Insomnia (ISI) ^c^	
	Median (IQR)	*rho*/*U*	Median (IQR)	*rho*/*U*	Median (IQR)	*rho*/*U*
Gender †						
Male	36 (7)	486,707 ***	12 (9)	473,586 ***	5 (8)	447,013 ***
Female	40 (10)		14 (9)		6 (9)	
**Age** (years) ‡		−0.08 **		−0.19 ***		−0.07 **
**No. in household**		0.007		0.03		−0.001
**Alcohol Consumption †**						
Medical Doctors						
Yes	38 (10)	61,635.5	14 (10)	72,137	6 (8)	74,254
No	39 (10)		14 (8)		5 (7)	
Nurses						
Yes	40 (12)	34,677	14 (9)	39,647	7 (9)	40,362
No	40 (11)		14 (9)		6 (9)	
Other HCPs						
Yes	39 (9)	30,189	13 (8)	35,875	5 (8)	37,079
No	40 (10)		13 (8)		4 (7)	
**Smoking †**						
Medical Doctors						
Yes	40 (10)	48,544 **	15.5 (10)	51,023 *	6 (9)	50,148
No	38 (9)		14 (10)		5 (8)	
Nurses						
Yes	43 (12)	31,725	15 (10)	34,700 *	7 (9)	32,458
No	40 (11)		13 (9)		6 (8)	
Other HCPs						
Yes	39 (10)	27,783	13 (8)	31,123	5 (9)	28,445
No	39 (9)		13 (9)		5 (8)	
**Gambling †**						
Medical Doctors						
Yes	39 (10)	12,049	14 (12)	11,296	6 (8)	13,119
No	38 (10)		14 (10)		5 (8)	
Nurses						
Yes	45 (13)	11,326 **	18 (9)	12,267 **	7 (11)	10,680
No	40 (11)		13 (9)		6 (8)	
Other HCPs						
Yes	42 (12)	8204 *	13 (8)	7254	7 (8)	8231
No	39 (9)		13 (9)		5 (8)	
**Hours Spent Online †**						
Medical Doctors						
Up to 2 h	38 (9)	89,466 ***	13 (10)	100,148 ***	4.5 (7)	100,002 ***
3 h or more	39 (9)		15 (9)		7 (8)	
Nurse						
Up to 2 h	40 (11)	398,66	12 (9)	47,862 ***	6 (9)	44,214 **
3 h or more	41 (12)		16 (9)		7 (9)	
Other HCP						
Up to 2 h	39 (9)	36,213	12 (8)	44,193 **	4	43,303 *
3 h or more	39 (10)		14 (8)		6	
Video games †						
Medical Doctors						
Yes	40 (10)	58,178 *	15 (9)	65,403 **	7 (9)	64,714 **
No	38 (9)		14 (10)		5 (7)	
Nurses						
Yes	43 (12)	30,330	15 (9)	33,661 *	7 (8)	32,771
No	40 (11)		13 (9)		6 (8)	
Other HCPs						
Yes	39 (11)	25,708	14 (7)	30,772	5 (8)	28,539
No	39 (9)		13 (9)		5 (8)	
Hours spent on video games †						
Medical Doctors						
<1 h	39 (9)	4336	16 (8)	4341	6 (9)	5085 *
≥1 h	40 (10)		15 (10)		8 (9)	
Nurses						
<1 h	41 (12)	3312	14 (7)	3834	7 (8)	3580
≥1 h	43 (12)		16 (10)		7 (8)	
Other HCPs						
<1 h	38 (11)	2558	13 (8)	2803	4 (8)	2860
≥1 h	39.5 (9)		14 (7)		6.5 (8)	

* *p* < 0.05; ** *p* < 0.01; *** *p* < 0.001; † Mann–Whitney U test; ‡ Spearman rank order correlation; ^a^ SAS, Zung’s Self-Rating Anxiety Scale; ^b^ PSS, Perceived Stress Scale; ^c^ ISI, Insomnia Severity Index.

## Data Availability

Data available upon request.
